# Robot-assisted autotransplantation of third molars in the maxilla: two case reports

**DOI:** 10.3389/froh.2025.1661873

**Published:** 2025-09-16

**Authors:** Yunkun Liu, Jia Song, Xiaoyu Chen, Chuyang Zhang, Yong Yang, Dan Liu, Haolin Zhou, Bingling Wu, Jian Zhang, Zhiyu Gu

**Affiliations:** Stomatology Hospital Affiliated to Zunyi Medical University, Zunyi, China

**Keywords:** dental autotransplantation, artificial intelligence, autonomous robotic surgery, 3D printing, tooth replica, oral surgery

## Abstract

Autotransplantation of teeth has attracted increasing attention due to its excellent biocompatibility and ability to preserve natural dentition. However, conventional autotransplantation of tooth techniques are highly technique-sensitive and reliant on clinician experience, limiting their predictability and broader clinical application. With the advancement of digital dentistry and surgical robotics, robot-assisted autotransplantation of teeth offers a new approach that enhances precision and consistency. In these two reports, digital intraoral scans and cone-beam computed tomography data were processed through AI-assisted segmentation, surgical path planning, and 3D printing technologies. Prior to robotic surgery, surgical guides were installed in the patient's oral cavity to perform calibration and ensure accurate alignment. During the procedure, an autonomous surgical robot was employed to prepare the recipient sites in the upper first molar regions. Following socket preparation, tooth replicas were used to simulate the transplantation process, allowing confirmation of fit and orientation before proceeding with the actual implantation. The transplantation of upper third molars was performed following a standardized digital protocol, involving one-time root canal treatment, 3 mm apicoectomy, and retrograde filling immediately after extraction. The treated teeth were then implanted into fresh extraction socket within the optimal time window, with the total duration from endodontic treatment to implantation not exceeding 15 min. The cases were followed up for three and six months, respectively, postoperative follow-ups showed favorable outcomes, including stable fixation, healthy surrounding soft tissues, and progressive bone healing as confirmed by radiographic imaging. These findings suggest that the integration of AI-based planning and robotic assistance significantly improves the predictability and clinical outcomes of autotransplantation of teeth, supporting its potential as a standardized and intelligent solution in modern dental surgery.

## 1 Introduction

As a crucial method for restoring partial edentulism, autotransplantation of teeth (ATT) has garnered increasing attention in clinical dentistry due to its superior biocompatibility and excellent recovery of physiological function (1). Compared to dental implants or other restorative approaches, ATT maximally preserves the biological characteristics of natural tooth while providing better clinical outcomes in terms of periodontal tissue integration, and aesthetics and ATT has broad indications and is applicable to a wide range of oral conditions, including developmental dental anomalies, congenital tooth agenesis (hypodontia), oroantral communications, alveolar clefts, deficient alveolar ridges, ectopic teeth, and maxillofacial injuries (2). However, traditional ATT requires a high level of surgical skill and experience from the operator (3). The minimally invasive extraction of donor tooth, precise preparation of the recipient site, and ensuring postoperative stability present significant challenges. Proper intraoperative preparation of the alveolar socket is essential for achieving optimal adaptation with the donor tooth, adequate postoperative stabilization and the patient's compliance with regular follow-up appointments are key determinants of successful ATT.

Compared to conventional surgical approaches, autonomous oral surgical robots, which integrate computer-aided design (CAD) and computer-aided manufacturing (CAM) technologies, represent a transformative advancement in dental surgery. These systems are capable of conducting preoperative 3D image reconstruction and precise surgical planning based on cone-beam computed tomography (CBCT) and intraoral scanning data, thereby enabling personalized and data-driven operative strategies (3). Intraoperatively, equipped with high-precision optical pose-tracking and real-time feedback systems, autonomous robots continuously monitor and visualize the surgical field, effectively eliminating blind spots and enhancing operational accuracy. Through intelligent control of robotic arms, these systems can autonomously perform key procedures such as alveolar socket preparation, donor tooth implantation, and recipient site optimization with sub-millimeter precision. This significantly reduces intraoperative variability caused by human factors and enhances overall surgical precision, safety, and minimally invasive performance. Additional safety mechanisms, including force feedback, emergency stop functions, and motion tracking, further safeguard the procedure by minimizing the risks of iatrogenic injury and over-manipulation (4).

These technological innovations contribute not only to improved initial stability of the transplanted tooth and favorable periodontal healing but also to higher long-term success rates of ATT. In recent years, the integration of artificial intelligence (AI) has propelled autonomous surgical systems toward greater autonomy and adaptability. AI-enabled surgical robots can simulate and analyze vast numbers of surgical cases, acquiring a database of experiential learning through high-throughput virtual procedures. This enables them to develop robust decision-making capabilities, adapt to inter-individual anatomical variation, and effectively manage complex clinical scenarios involving comorbid conditions (5).

**Figure 1:**
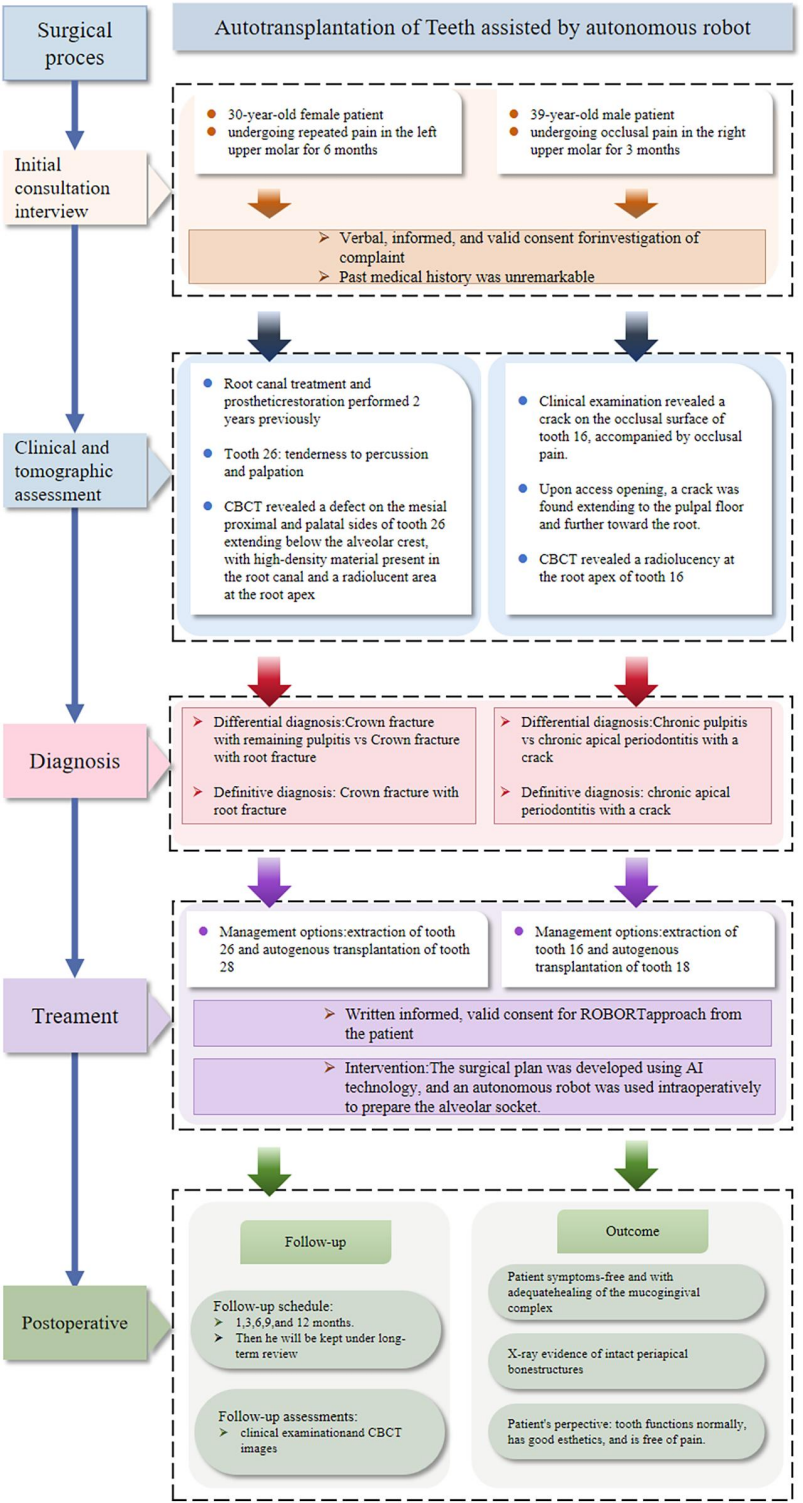
Timeline of events describing the flowchart of the cases.

Moreover, autonomous surgical robots are generally expected to reduce surgeon fatigue, shorten operative time, and minimize complications. However, these advantages may vary depending on the specific clinical context. These features are especially valuable in procedures like ATT, where surgical precision and tissue preservation are critical. Collectively, these advancements are paving the way for the standardization, digitization, and intelligent evolution of ATT, establishing a new paradigm for precision oral surgery (Figure 1) (6).

This case report aims to demonstrate the feasibility and clinical performance of autonomous robotic assistance in ATT, with particular attention to surgical accuracy, operative time, and postoperative healing.

## 2 Cases presentation

### 2.1 Case 1

**Figure 2:**
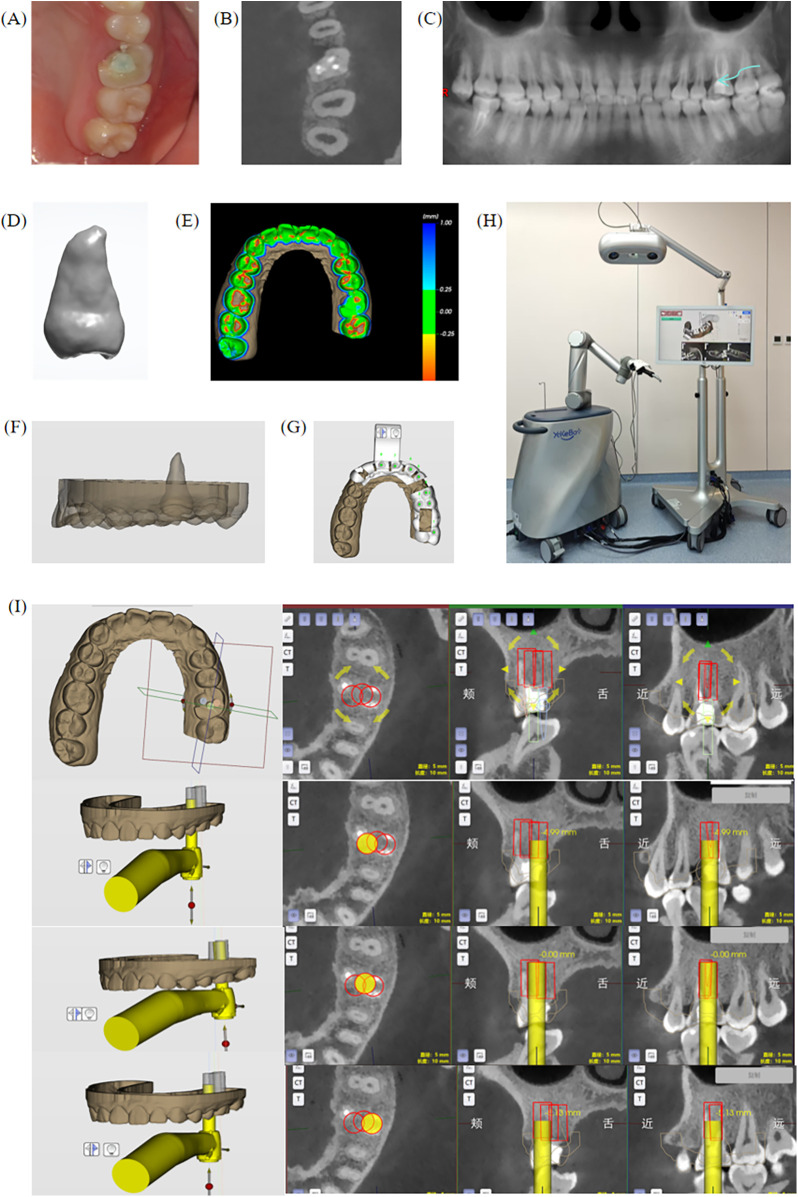
Clinical, imaging examination and preoperative software design of case 1. **(A)** Clinical examination. **(B,C)** Radiographic examination, showing the palatal fracture extending below the alveolar crest. (d) AI simulation image of donor tooth 28. **(E)** CBCT and intraoral scan data registration and alignment, showing differences. **(F)** 3D simulation showing buccal view of tooth 28 implantation. **(G)** Surgical guide design. **(H)** An autonomous surgical robot. **(I)** Robot software design and 3D simulation of three drill paths for alveolar socket preparation and the intraoperative drilling approach and positioning from the palatal side, central, and buccal side using the mobile drilling tool.

The first patient was a 30-year-old female who had experienced repeated pain in the left upper molar for six months.She reported that two years ago, root canal treatment had been performed on the left upper molar due to a crown fracture. Subsequently, due to persistent pain on occlusion, root canal retreatment was performed. However, the patient continued to experience recurrent occlusal pain in the upper left posterior tooth over the past six months and subsequently sought diagnosis and treatment at our department. The patient had been in good general health and denied any systemic diseases. Clinical examination revealed a temporary filling over a defect on the palatal side of the occlusal surface of tooth 26, with the fracture extending under the gingiva, percussion test (++), no mobility and no gingival redness or swelling (Figure 2A). CBCT revealed a defect on the mesial proximal and palatal sides of tooth 26 extending below the alveolar crest, with high-density material present in the root canal and a radiolucent area at the root apex (Figures 2B,C). Tooth 28 appeared as a conical tooth.

Based on the clinical and CBCT findings, a diagnosis of tooth 26 coronal defects (after root canal retreatment/suspected root fracture) was concluded, and tooth 28 was completely erupted, whereas tooth 38 showed no occlusal contact with tooth 28. The patient was no longer willing to endure the pain and strongly requested to have the tooth extracted, then the patient opted for extraction of tooth 26 and autotransplantation of tooth 28.

DentalNavi is a powerful digital surgical planning software that integrates preoperative design, tooth segmentation, and intraoperative simulation-based registration. It was the sole software used throughout the entire preoperative workflow, demonstrating its efficiency and practicality in intelligent dental surgery. Using the DentalNavi software, patient information was entered, and DICOM-format CBCT data was imported. A panoramic curve was added, and a manual threshold segmentation model was applied, followed by AI-assisted segmentation of the donor tooth 28. Standard tessellation language (STL) data were then generated and further processed using surface smoothing and gap filling techniques to optimize the 3D model for subsequent surgical planning and 3D printing (Figure 2A). A simulation of the donor tooth 28 showed the positional relationship between the donor tooth and the recipient site and determined the implantation depth, height, and angle. The software's “Occlusal Relationship Display” function was used to adjust the occlusion of the donor tooth, ensuring accurate occlusal positioning (Figure 2F). Based on the simulated analysis in the planning software, tooth 28 was deemed the most suitable donor for transplantation to the site of tooth 26. This choice allowed for optimal adaptation with minimal alveolar bone preparation. Additionally, since both teeth are located on the same side, postoperative masticatory function would be less affected, which is favorable for wound healing. Moreover, tooth 28 had no functional occlusal contact with the opposing tooth 38, further supporting its selection as the donor tooth. A surgical guide was then generated (Figure 2G). During surgery, a 5 mm pineapple drill bit was used, and the software was utilized to plan the alveolar socket preparation depth, angle, and optimal position in the mouth to avoid damaging adjacent tooth or soft tissues. The robotic motion used in this procedure, as in dental implant cases, was single-axis. So the simulation images in the software revealed partial overlap among the three pineapple drill trajectories, effectively ensuring that the prepared alveolar socket conformed to the donor tooth's dimensions in length and width (Figure 2I). This approach also minimized the number of robotic drilling steps, reduced the frequency of drill bit changes, and shortened the total preparation time.

**Figure 3:**
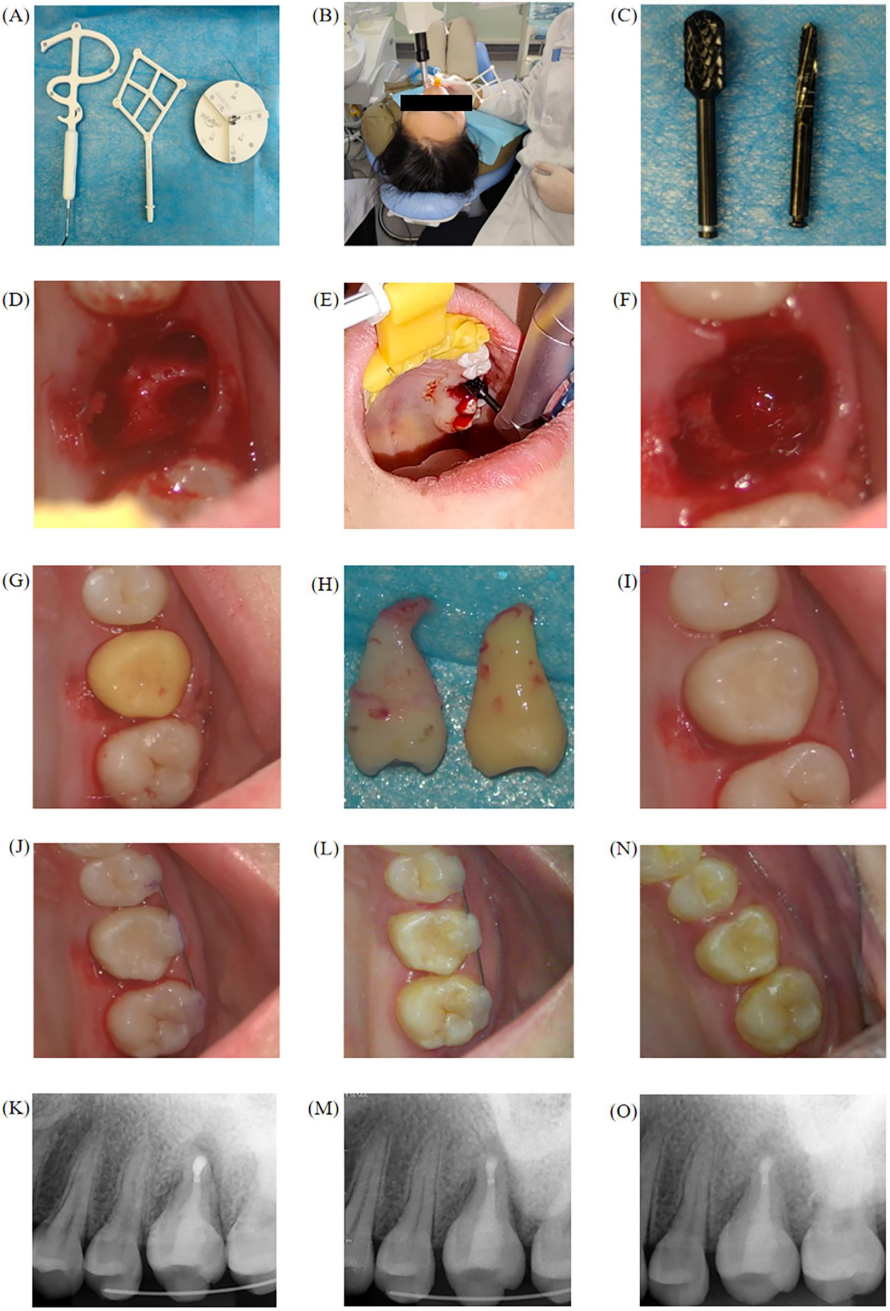
Surgical procedure for case 1. **(A)** Registration accessories. **(B)** Calibration process. **(C)** The drill bit used during the procedure. **(D)** Alveolar socket morphology after extraction of tooth 26. **(E)** The robot begins preparing the alveolar socket for tooth 26. **(F)** Alveolar socket preparation completed by the robot. **(G)** 3D-printed tooth trial implantation, with proper fit. **(H)** Tooth 28 extracted and compared with the 3D-printed replica for morphology. **(I)** Tooth 28 implantation. **(J,K)** Clinical and imaging examination of case 1 immediately postoperatively. **(L,M)** Clinical and imaging examination of case 1 at the first month postoperatively. **(N,O)** Clinical and imaging examination of case 1 at three month postoperatively.

Preoperative preparation for the autonomous robot was performed (Figure 2H). The system automatically completed robot end-effector calibration and needle tip calibration to obtain the proper positioning of the alveolar socket preparation tool. The surgical guide was first secured onto the patient's teeth, and visual markers were then attached to the surgical guide, and calibration probes were used for calibration (Figures 3A,B). Routine disinfection and draping were performed, the corresponding drill bit was installed on the mobile drill (Figure 3C), followed by a local anaesthetic block for extraction of tooth 26 (Figure 3D), and the autonomous robot followed the planned path to complete the alveolar socket preparation (Figure 3E). During the preparation process, any positional errors could be observed on the Multi-plannar reconstruction (MPR) display. If a deviation in the surgical position occurred, the drill bit colour on the MPR view would change, and the mobile drill would stop to ensure safety. The surgical path would then be re-planned. Once the fresh extraction socket preparation was completed (Figure 3F), a 3D-printed replica of tooth 28 was placed in the recipient site for trial implantation (Figure 3G), confirming the fit between the socket's size and depth and the replica. Subsequently, tooth 28 was extracted (Figure 3H), followed by root canal therapy, 3 mm apical resection, reverse preparation, and use of C-Root BP to reverse filling performed ex vivo. The extra-alveolar time, starting from the extraction of the donor tooth, was 11 min in total, encompassing extraoral root canal treatment and successful implantation into the recipient site. The tooth was then implanted into the recipient site (Figure 3I). Flowable resin and wire were used for buccal fixation of tooth 25–27, and the gingiva in the surgical area was sutured using 3–0 non-resorbable sutures. Slight adjustments were then made to the occlusion of the donor tooth. Postoperative instructions were given according to standard tooth extraction protocols, advising the patient to rest following extraction care guidelines. The patient was allowed to leave the clinic only after biting on a cotton roll for 30 min without active bleeding. Additionally, detailed explanations regarding the use of antibiotics were provided to the patient.

Removal of the splint was performed one month postoperatively. Three months after the surgery, a follow-up examination revealed that the gingival healing at the transplant site was good, the donor tooth showed no significant mobility, and no complications reported, and the patient expressed high satisfaction with the outcome (Figures 3J–O).

### 2.2 Case 2

**Figure 4:**
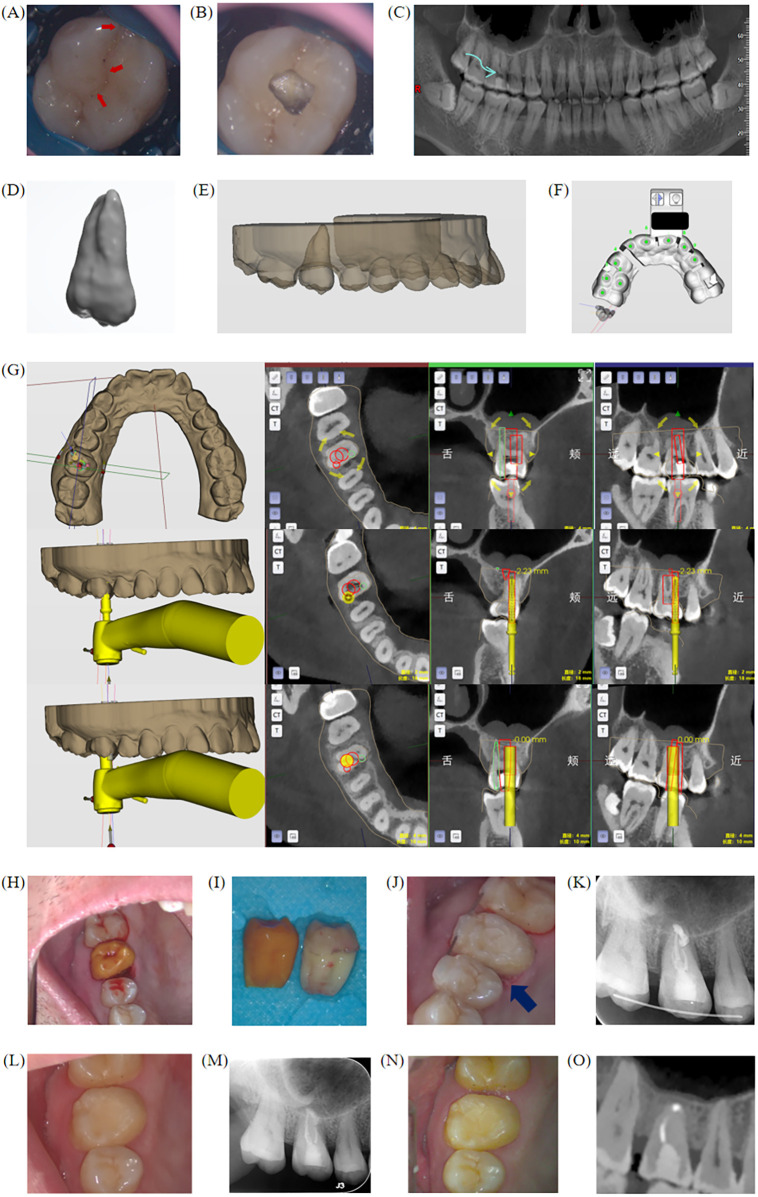
Clinical, imaging examination and preoperative software design of case 2. **(A,B)** Occlusal surface showing the visible crack, with the crack extending to the pulp chamber floor after access. **(C)** Radiographic examination showing a radiolucency at the root apex. **(D)** AI simulation image of donor tooth 18. **(E)** 3D simulation showing buccal view of tooth 28 implantation. **(F)** Surgical guide design. **(G)** Software design and 3D simulation of four drill paths for alveolar socket preparation. **(H)** Trial implantation of 3D-printed tooth 18 after extraction of tooth 16, with proper fit. **(I)** Tooth 18 extracted and compared with the 3D-printed replica for morphology. **(J)** Immediate postoperative intraoral photo. **(K)** Immediate postoperative x-ray. **(L,M)** Clinical and imaging examination of case 2 in the fourth month postoperatively. **(N,O)** Clinical and imaging examination of case 2 in the sixth month postoperatively.

The second patient was a 39-year-old male who had experienced occlusal pain in the right upper molar for 6 months. The patient had been in good general health and denied any systemic diseases. Clinical examination revealed a crack on the occlusal surface of tooth 16, extending from the mesial to the distal and crossing the marginal ridge (Figures 4A,B). Percussion test (++), no mobility, and slight gingival redness and swelling were noted. panoramic radiograph revealed a radiolucency at the root apex of tooth 16 (Figure 4C). Tooth 16 was diagnosed with chronic apical periodontitis with a crack. Upon opening the pulp, the crack was found to extend to the pulp chamber floor and spread to the root direction. Therefore, the patient opted for autogenous tooth transplantation. Teeth 38 and 48 were mesially impacted and presented significant difficulty and risk for complete extraction, making them unsuitable as donor teeth. In contrast, tooth 18 was located on the same side as tooth 16, which helped minimize the number of surgical sites in the oral cavity, thereby promoting postoperative recovery and reducing surgical trauma. Compared to tooth 28, tooth 18 was considered a more appropriate donor tooth in this case. After discussing treatment options with the patient and considering the long-term prognosis of the tooth, the patient chose extraction of tooth 16 and autogenous transplantation of tooth 18. The software design process followed the same steps as in case 1, where a pilot drill and a circumferential drill were selected as the surgical drill bits, and the surgical path was planned (Figures 4D–G). After extraction of tooth 16, the alveolar socket was prepared using the autonomous robot. Unlike Case 1, due to the choice of drill bit in this case, some sharp areas or partially prepared regions remained, which required manual smoothing with a surgical handpiece during the trial implantation of the 3D-printed tooth 18, the trial implantation was attempted a total of three times to ensure optimal fit and stability (Figure 4H), confirming the socket's fit. Tooth 18 was then extracted (Figure 4I) and underwent root canal treatment, apical resection, and reverse filling ex vivo. The surgical area was thoroughly irrigated with saline, the donor tooth was implanted, fixed, and using 3-0 non-resorbable sutures, occlusal adjustments, and immediate postoperative x-rays were taken (Figures 4J,K). Postoperative instructions were given according to standard tooth extraction protocols. The extra-alveolar time, starting from the extraction of the donor tooth, was 14 min in total, encompassing extraoral root canal treatment and successful implantation into the recipient site. The splint was removed after one month. At the 4-month follow-up (Figures 4L,M) and 6-month follow-up (Figures 4N,O), percussion of the donor tooth was negative (-), with normal physiological mobility, good gingival healing, and normal alveolar bone height visible on the x-ray. No significant bone resorption was observed at the root apex or surrounding periodontal area, and no complications reported, and the patient expressed high satisfaction with the outcome.

## 3 Discussion

ATT involves relocating a patient's own tooth to another site within the oral cavity to restore missing dentition. Common donor teeth include third molars, fully developed impacted teeth, supernumerary teeth, and premolars scheduled for extraction due to orthodontic indications. These donor teeth are often used to replace congenitally missing teeth or teeth lost due to trauma or caries (4). Compared to dental implants, ATT offers superior biocompatibility and does not cause immune rejection (5). Therefore, ATT is a promising therapeutic strategy for tooth loss restoration.

Various factors, including the donor tooth's developmental stage, the recipient site's supporting bone conditions, and postoperative care, influence the success rate of ATT. Therefore, accurate preoperative evaluation, optimized surgical techniques, and standardized postoperative management are crucial for improving the success rate of transplantation. Among these factors, selecting the donor tooth is critical, and tooth with incompletely developed roots are typically preferred. According to the Demirjian (6) classification system, tooth development is divided into three stages: A-D for crown development completion, E-F for root formation beginning with open apices, and H for fully developed and closed root apices. Research shows that incompletely developed roots have a 98% survival rate one-year post-transplantation and survival rates of 97%, 95.5%, and 96.9% at two, five, and ten years, respectively. In contrast, when the donor tooth is in the F developmental stage, transplantation success rates are higher (87.5%) than for tooth in the E or G stages (7, 8). Consequently, the root apex's continued development is key to improving transplantation survival rates. Direct transplantation is recommended for donor's tooth with incompletely developed roots, avoiding prophylactic root canal treatment or apex resection to allow further root development. This procedure can also preserve pulp vitality after transplantation, promoting further root development and a more natural physiological reconstruction (9). Although transplantation of donor teeth in Stage F has been associated with higher success rates than those in Stages E or G, it is important to note that autotransplantation of teeth with underdeveloped roots (Stage E) can also yield favorable outcomes due to their potential for continued root development (10).

For fully developed roots, preserving the periodontal ligament's integrity is crucial (11). The stem cells and growth factors in the periodontal ligament promote alveolar bone reconstruction, reducing root surface resorption and alveolar bone loss after transplantation, improving long-term stability, and forming typical periodontal ligament structures, making the donor tooth function more like a natural tooth. Third molars are often used as donor tooth with complex root canal systems, especially significant branching within the last 3 mm of the root apex. Even after thorough root canal treatment post-transplantation, achieving a complete, tight seal remains difficult, increasing the risk of transplantation failure (12). Therefore, for patients with fully developed roots, establishing a multidisciplinary team capable of performing root canal treatment and apex resection concurrently during the ATT process while keeping the ex vivo time under 15 min may improve long-term transplant retention. However, further clinical research is needed to confirm (13).

In the 21st century, new generations of surgical robots equipped with high-resolution imaging systems, enhanced visual capabilities, and multi-degree-of-freedom precision tools have expanded their clinical applications. AI can construct 3D anatomical models based on CT, MRI, and ultrasound images, assisting surgeons in precise personalized surgery planning (14). AI, trained on large-scale surgical data, can optimize operative paths and provide intelligent decision support, enhancing surgical efficiency and stability (15, 16). With advances in AI and robotics, AI-driven surgical robots are expected to further promote intelligent and precise surgery by integrating computer vision, deep learning, real-time data analysis, and automation, thereby improving precision, reducing errors, and enhancing safety (17, 18).

In oral surgery, surgical robots are widely applied in implant surgery. Yang et al. (19) conducted a systematic review and meta-analysis evaluating the precision of implant surgery assisted by robotic systems, demonstrating that robotic systems help reduce angular deviations in implant placement. Similarly, Zhang et al. demonstrated the high precision of robot-assisted techniques in bone windowing and tooth extraction, while also pointing out limitations such as increased economic burden and a prolonged learning curve (20). The application of surgical robots in endodontic therapy is gradually expanding, covering root canal cleaning, shaping, and surgical operations. Recent studies have highlighted the clinical potential of autonomous robotic-assisted systems in various complex dental procedures. The Isufi team (21) used a passive dental implant robot system (Yomi) to assist with a microscopic apical surgery of a left upper premolar. However, the system requires physician collaboration and cannot perform the surgery autonomously. Gong et al. successfully applied an autonomous robot to perform microsurgical apical surgeries on anatomically complex first molars, effectively avoiding the risks associated with freehand operation, such as inaccurate positioning, excessive bone removal, and damage to adjacent structures (22). Qin et al. (23) completed root canal retreatment of the lower second molar using robotic assistance, precisely removing fibre-like blockages from the root canal. Wang et al. reported the safe and effective use of an autonomous robotic system for the removal of a fiber post during root canal retreatment of a first molar, significantly reducing the risk of intraoperative deviation and perforation (24). Furthermore, Fu et al. employed robotic assistance to precisely localize and retrieve a separated instrument beyond the apical foramen while preserving cortical bone integrity and minimizing bone removal. Collectively, these findings suggest that autonomous robotic systems offer promising advantages in terms of precision, safety, and minimally invasive operation, particularly in anatomically challenging or high-risk endodontic and surgical cases (25). Despite the significant potential of robots in endodontic therapy, practical implementation faces challenges such as high costs, technical limitations, and lack of haptic feedback. Therefore, it is recommended that long-term clinical research and research and development investment be strengthened to promote the broader clinical application of robotic technology in endodontic therapy (26).

ATT initially relied on traditional free-hand surgery, depending on the operator's experience with implantation. However, this approach leads to significant errors in implant angle and may damage adjacent anatomical structures. With the development of digital guides and dynamic navigation technology, intelligent autonomous robotic technology has revolutionized the ATT, pushing the field toward human-robot collaboration. Recently, a new autonomous oral implant robot with AI, developed by a team led by Academician Zhao Yimin from the Fourth Military Medical University, has upgraded its visual system to infrared vision, reducing marker object size while enhancing visual system stability. The multi-functional control system enables the robot to perform various complex procedures, including implant placement (21, 27), microscopic apical surgery (28), complete extraction of fully impacted tooth, and ATT (29). Several studies have shown that compared to free-hand surgery, static guides, and dynamic navigation methods, autonomous robots demonstrate superior accuracy in crown-root angle deviation control, further validating their potential clinical value in ATT (19, 30). Furthermore, compared to the traditional free-hand technique, the precision of robot-assisted technology and dynamic navigation has been significantly validated in endodontic surgery (31, 32).

Although autonomous surgical robots offer significant advantages in surgical functionality, minimal invasiveness, and precision, their widespread clinical application faces challenges due to high development and implementation costs. Firstly, the robot's operating interface is relatively complex, and physicians face a steep learning curve when mastering the system's operations and functionalities, limiting its clinical adoption. Therefore, optimizing the robot software interface, simplifying operational workflows, and reducing redundant steps will help enhance the system's usability and user-friendliness, reducing the difficulty for physicians to master it. With the continuous advancement of medical technology, incorporating surgical robotics into the training of interns and clinical practitioners is of significant importance. Such integration not only facilitates the widespread adoption of innovative techniques but also contributes to the standardization and precision of clinical procedures. In recent years, mixed reality (MR) and immersive reality (IR) technologies have demonstrated considerable potential in oral and maxillofacial surgical training. By simulating realistic surgical environments, these technologies enhance spatial awareness and operational proficiency, shorten the learning curve, and improve the interactivity and safety of training programs. As a result, they offer valuable educational support, particularly for complex surgical procedures (33, 34). In addition to the above limitations, several practical challenges must also be addressed to facilitate the broader clinical integration of autonomous surgical robots. These include the prolonged and complex preoperative preparation process, the necessity for system calibration, and the continued dependence on human intervention for key intraoperative steps—such as the placement or repositioning of the surgical guide, donor tooth replica, and drills. Furthermore, in certain cases, manual refinement of the recipient socket using a surgical handpiece may be required to accommodate anatomical variability or correct incomplete preparation by the robotic system. These limitations highlight the current barriers to fully autonomous operation and underscore the need for further technical refinement and workflow optimization. Secondly, establishing a systematic short-term training course and standardized assessment and certification system will allow healthcare workers to quickly acquire the key operational skills and troubleshooting strategies, shortening the learning period and promoting the widespread clinical adoption of this technology. Thirdly, the preoperative preparation process for autonomous surgical robots is complex and requires a professional team to coordinate all steps. Controlling intraoperative time is critical, especially in ATT, where the donor tooth's ex vivo time needs to be minimized. Emerging evidence suggests that intraoperative application of 3D-printed replicas facilitates precise evaluation of the alveolar socket preparation and optimal donor tooth positioning, which contributes to a reduction in extra-alveolar time and potentially enhances the overall success rate of tooth autotransplantation (35). Optimizing the robotic preoperative preparation process and improving team collaboration efficiency are essential for the clinical application of this technology. Finally, there is currently no suitable drill for ATT during socket preparation. We have improved the combination of preparation drills by selecting a 4–5 mm pineapple drill, reducing the number of preparation steps from 4 to 6 to 2–3 times, significantly shortening the surgery time. In the future, our team's goal is to design a personalized ATT drill highly compatible with autonomous surgical robots, ensuring precise socket preparation, improving cutting efficiency, reducing bone tissue damage risk, and advancing the development of ATT.

The autonomous surgical robot used in this report is capable of multi-axis movement and has been validated in preclinical experiments, the two clinical cases reported herein employed a single-axis drilling mode. This decision was made based on safety considerations, as single-axis drilling—along the predefined axial direction—offers greater procedural stability and has been widely reported in the literature to be safe and effective for dental applications. While multi-axis capabilities provide enhanced flexibility for addressing complex anatomical variations, their clinical implementation requires further validation. Future studies will explore the clinical application of the robot's multi-axis functions to assess their feasibility, safety, and potential advantages in more anatomically challenging scenarios.We referred to relevant literature on extraoral root canal treatment and adopted this approach during the surgery, ensuring that the duration from root canal treatment to implantation was controlled within 15 min (36). Nevertheless, we acknowledge the potential concerns associated with extraoral procedures, such as periodontal ligament viability, and plan to conduct future studies comparing intraoral and extraoral RCT approaches in the context of autotransplantation.

ATT is a promising option for tooth replacement. Robotic-assisted techniques—including AI-based segmentation, 3D planning, and precise intraoperative execution—enhance surgical accuracy, stability, and workflow efficiency. However, this report is limited by the small number of cases, short follow-up periods, and certain procedural constraints such as single-axis drilling and extraoral root canal treatment. Further studies with larger cohorts and long-term follow-up are needed to confirm these preliminary findings and support broader clinical application.

## Data Availability

The raw data supporting the conclusions of this article will be made available by the authors, without undue reservation.
